# Application of Neutral Electrolyzed Water on pork chops and its impact on meat quality

**DOI:** 10.1038/s41598-020-76931-4

**Published:** 2020-11-16

**Authors:** Erwin Torres-Rosales, Andres Rivera-Garcia, Patricia Janet Rosario-Perez, Juan Carlos Ramirez-Orejel, David Paez-Esquiliano, Sandra Martinez-Vidal, Eduardo Guzman-Olea, Jose Alberto Cano-Buendia

**Affiliations:** 1 Facultad de Medicina Veterinaria y Zootecnia, Department of Microbiology and Immunology, Universidad Nacional Autónoma de Mexico (UNAM), 04510 Mexico City, México; 2 Facultad de Medicina Veterinaria y Zootecnia, Department of Animal Nutrition and Biochemistry, Universidad Nacional Autónoma de México (UNAM), 04510 Mexico City, Mexico; 3 Facultad de Medicina Veterinaria y Zootecnia, Department of Physiology and Pharmacology, Universidad Nacional Autónoma de México (UNAM), 04510 Mexico City, Mexico; 4 Esteripharma S.A. de C.V., 50450 Atlacomulco, Estado de Mexico Mexico; 5 CONACYT-Universidad Autónoma del Estado de Hidalgo, Pachuca, Hidalgo Mexico

**Keywords:** Drug discovery, Microbiology, Health care

## Abstract

Physicochemical and microbiological properties of pork chops sprayed with Neutral Electrolyzed Water (NEW) were evaluated during storage at refrigeration temperature. Pork chops were randomly allocated into three groups and were artificially contaminated with an inoculum of 10^6^ CFU/mL of *Listeria monocytogenes*. Each group was treated with either NEW (58 ppm), NaClO (35 ppm), or saline solution (SS). Subsequently, recovered bacteria were plated on TSA petri dishes and the reduction percentage of *Listeria monocytogenes* was calculated 24 h and 8 days after treatment. Physicochemical analysis [pH, content of lactic acid, thiobarbituric acid reactive substances (TBARS) and total volatile base nitrogen (TVBN)] were performed to evaluate the effect of all solutions used on pork meat kept at 4 °C for 19 days. In vitro NEW reduced *L. monocytogenes* titers by > 99.98% and 80.19% and 90.35% in artificially contaminated pork 24 h and 8 days after NEW treatment, respectively. Compared to the SS treatment, NEW and NaClO solutions caused a 0.67 Log UFC/g and 0.65 Log UFC/g reduction respectively. After eight days post-treatment, NEW and NaClO bacterial titers were below the SS treatment. NEW caused little color change in treated meat. It helped to reduce the formation of lactic acid and TVB-N when pork chops are kept at 4 °C for 19 days. Therefore, NEW could be considered as a new alternative to sanitize and preserve pork meat.

## Introduction

Foodborne illnesses are a major concern in the food industry. Pork is one of the most important and widely produced types of meats worldwide^1^. In order to produce and process large amounts of pork, intensive systems have been developed but preservation methods need to be improved because the meat and its sub-products are perishable. Many different solutions have been developed to decrease the microbial contamination of meat^2^. Chemical solutions like organic acids or chlorine are used worldwide to maintain meat quality. However in Belgium, Denmark, Germany, the Netherlands, and Switzerland the use of sodium hypochlorite (NaClO) in the food industry is banned^3,4^. Moreover, the use of chemical solutions could be problematic since it can affect physical or chemical properties of the meat or carcasses like the color, smell or texture^5^. These attributes are important to customers in determining the acceptability of their meat^6^ and, as a consequence, shelf life can be modified since meat color can be influenced by the onset of oxidation during refrigerated storage^7^ or contamination with food borne pathogens like *Listeria monocytogenes*. Outbreaks or recalls have been reported in contaminated pork with *L. monocytogenes*^8,9^*;* however, small bacterial loads can be difficult to identify in the beginning and a lack of temperature control could contribute to develop outbreaks^10^. New technologies and chemical solutions have been developed to eliminate microbial pathogens from carcasses or meat. One alternative is the use of Electrolyzed Water (EW). EW has been used in different ready to eat products like lettuce^11^, spinach^12^, and strawberries^13^. EW has many advantages; it does not harm human surfaces like the mucosa^14^, stainless steel (only neutral EW)^15^, or eggshell cuticles^16^, and it does not have a negative impact on the environment^17^ since it reverts to normal water and Na^+^/Cl^−^ ions after use^17,18^. In addition, it has been reported that its use does not affect physicochemical and sensory characteristics of chicken meat^19^. Neutral Electrolyzed Water (NEW), a different version of EW, has an oxidation reduction potential (ORP) of 750–900 mV^16,20–23^ and its main component is hypochlorous acid (HOCl)^24^.

The goal of this study was to evaluate the bactericidal effect of highly concentrated NEW when it is sprayed on artificially, highly contaminated pork chops with *L. monocytogenes* or *Salmonella* Typhi*,* without affecting meat´s physicochemical properties (color, pH, lactic acid concentration, and lipid oxidation) and quality (total volatile base nitrogen).

## Material and methods

### Bacterial strain and inoculum preparation

*Listeria monocytogenes* and *Salmonella* Typhi were obtained from the American Type Culture Collection (ATCC 19115 and ATCC 9992 V). *L. monocytogenes* was grown in Brain Heart Infusion agar (Bioxon, Cat. No. 214700, Estado de Mexico, Mexico) at 37 °C for 24 h. Gram staining^25^ and *Salmonella* Typhi was grown using Salmonella Shigella agar (MCD LAB, Cat. No. 716, Estado de Mexico, Mexico) the Vitek2 system (BioMérieux Cat. No. 27630, Marcy-l´Étoile, France) was used as confirmative method. A single colony of *L. monocytogenes* or *Salmonella* Typhi was grown in 50 mL of Trypticase Soy Broth (TSB) (BD Bioxon, Cat. No. 211670, Mexico, Mexico) at 37 °C for 16 h. Determination of viable cells was conducted according to the Mexican Official Norm for aerobic plate counting^26^. Decimal serial dilutions were performed in PBS for a final volume of 10 mL. One hundred microliters of each dilution were plated on a petri dish containing 15 mL of trypticase soy agar (TSA) (MCDLAB, Cat. No. 7171, Estado de México, Mexico). The plates were incubated overnight at 37 °C and plate counting was performed.

### Evaluation of solutions

Neutral Electrolyzed Water (NEW) was provided by Esteripharma S.A. de C.V. The concentration of sodium hypochlorite (Quimica Rique, Cat. No. 7681-52-9, Estado de Mexico, Mexico) was adjusted to 35 ppm since that is below the maximum allowed concentration for reed meat carcasses and contaminated chicken carcasses^27^; this was used as a disinfectant control. Saline solution (SS) (NaCl, Cat. No. 6845, Meyer, Mexico) was prepared as a wash control. The pH and ORP values were measured using a portable pH/ORP/temperature combo tester (Cat. No. HI98121, Hanna Instruments, Rhode Island) following the manufacturer instructions. Chlorine concentration was measured using a chlorine portable photometer (Cat. No. HI96771, Hanna Instruments, Rhode Island) and the iodometric method was used^28^ to evaluate free chlorine content.

### Bactericidal in vitro test

The Mexican Norm NMX-BB-040-SCFI-1999 was performed. Briefly described, all working solutions were evaluated (NEW, NaClO and SS) then 99 mL of each tested solution was transferred to a sterile 250 mL Erlenmeyer flask with a screw cap. Flasks were shaken and before the liquid stopped moving, 1 mL of *L. monocytogenes* or *Salmonella* Typhi inoculum was added to facilitate its incorporation. After 30 s, 1 mL of the mixture was transferred to a tube containing 9 mL of 0.1% peptone water (used as neutralizing solution) and subsequently mixed. Decimal serial dilutions were performed, and 1 mL aliquots of each dilution was plated on petri dishes containing TSA. Plates were incubated for 48 h at 37 °C. After the incubation period, the number of CFU was counted.

### Pork chops collection and allocation

Pork chops were obtained from the Center of Teaching, Research and Extension in swine production (CEIEPP) at the Autonomous National University of Mexico. The pig breed was a mix of York-Pietrain and Duroc-Landrace. All samples were kept at 4 °C for 24 h before use. Pork chops were cut into approximately 150 g pieces, after, 90 pieces were weighed, kept in plastic bags (Nasco Whirl–Pak, B01065WA, Fort Atkinson, WI), and kept at 4 °C until further use.

### Contamination of pork chops

Inoculum was prepared using an overnight culture of *L. monocytogenes* or *Salmonella* Typhi*.* Contamination inoculum was prepared using 0.1% peptone water adjusting the bacterial count to 10^6^ CFU/mL. The inoculum was kept in a plastic container within a laminar flow safety cabinet (NUAIRE UN-440-400). Pork chops were divided in two groups; first group of 45 pieces were submerged into *L. monocytogenes* inoculum for 15 min and were kept on sterile plastic colanders for 5 min to let the inoculum drain from the meat into a biosafety cabinet at room temperature^5^. The second group of pork chops was submerged into *Salmonella* Typhi inoculum and handled using same methodology as the first group.

### Treatment of pork chops

Artificially contaminated pork chops were divided into three groups containing 15 pieces each. Groups were labeled as NEW, NaClO, and SS. Working solutions were applied using spray bottles containing 15 mL of each solution to each group. The meat was turned over when half of the treatment was applied, and the remaining solution was used. Working solutions were in contact with pork samples for 60 s. Pork chops were individually deposited into a plastic bag containing 100 mL of 0.1% peptone water to collect the surviving bacteria after treatment. The meat was hand rubbed for 1 min and 1 mL aliquots were taken from the plastic bags and used for plate counting. Pork chops were individually placed into new plastic bags and kept at 4 °C. Bacterial collection and tittering were performed at days 1 and 8.

Non-contaminated pork chops were also divided into three groups containing 15 pieces each. They were treated with the evaluated solutions as it was described above. After treatments, meat was kept in individual plastic bags at 4 °C. Samples or readouts were taken on days 1, 3, 5 12 and 19 after each treatment for physicochemical analysis [pH, content of lactic acid, thiobarbituric acid reactive substances (TBARS) and total volatile base nitrogen (TVBN)] and total aerobic viable counts were determined using TSA plates at days 1 and 8.

### Analysis of bactericidal effect on pork meat

Aliquots from days 1 and 8 from artificially contaminated pork and non-contaminated samples were used to calculate the bacterial titer using the most probable number methodology. Obtained colonies were counted, adjusted by the dilution factor, and reported as CFU/g. The percentage of reduction in bacterial titer from all treatments was calculated using Eq. (1):

1 $$Porcentage of reduction=\frac{\left(\frac{CFU}{g}SS - \frac{CFU}{g}Treatment\right)}{\frac{CFU}{g}SS}*100$$

where SS is the mean saline solution treatment titer and treatment is the mean titer of NEW or NaClO treatments.

### Color measurements

To obtain color characteristics before and after treatments, a spectrophotometer (Konica Minolta CM-600d, Ramsey, NJ) was used. Five random zones were used to measure *L*, *a,* and *b* parameters that compose the color space (*CIELab*). The ΔE value from each treatment was calculated using Eq. (2):

2 $$\Delta E=\sqrt{({{L}_{2}-{L}_{1})}^{2}+({{a}_{2}-{a}_{1})}^{2}+({{b}_{2}-{b}_{1})}^{2}}$$

where 1 is the value before treatment and 2 is the value after treatment with the specific evaluated solution. Color parameters were measured at days 0, 1, 5, 12 and 19.

### pH measurement

The Mexican Norm^29^ was used to obtain the pH of samples after treatment. Briefly, 10 g of sample was blended with 100 mL of sterile distilled water. A portion of the prepared sample was placed into a beaker and stirred. Temperature was adjusted to 20 °C ± 0.5 °C and pH value was obtained using a pH meter.

### Determination of lactic acid

The methodology described in the Mexican Norm NMX-F-102-NORMEX-2010^30^ was used. In brief, 10 g of sample was added to a blender and 100 mL of sterile distilled water then added. The formed paste was filtered using grade 4 filter paper (Whatman, Cat. No. 1004-917, NJ) and the diluted sample was transferred to an Erlenmeyer flask. Samples were stirred manually and 250 μL of 1% phenolphthalein (Sigma Aldrich, Cat. No. 105945, MO) was added. Samples were then titrated using 0.1 N sodium hydroxide solution (Sigma Aldrich, Cat. No. 795429, MO), until it turned pink. Titrations were repeated without pork meat and results were compared using the Eq. (3):

3 $$\mathrm{lactic acid }(\mathrm{\%})\left(\frac{\mathrm{ lac ac }(\mathrm{g})}{100\mathrm{ mL}}\right)=\frac{\left(\mathrm{A}-\mathrm{B}\right)*\mathrm{C }*\mathrm{MM}}{\mathrm{pork }(\mathrm{g})}*100$$

where A − B is the Corrected volume [NaOH sample (mL) − NaOH control (mL)], C is the Concentration of NaOH and MM is the Molar Mass of lactic acid (90 g/mol).

### Determination of thiobarbituric acid reactive substances (TBARS)

TBARS were obtained by the methodology reported by Hernández et al.^31^. Briefly, 25 g of pork with 100 mL of 5% trichloroacetic acid (Sigma Aldrich, Cat. No. T63399, MO) was blended and then centrifuged at 10,000 rpm for 20 min. The supernatant was filtered using filter paper Whatman type 4. After, 2 mL of the filtrate was transferred to a glass tube and 2 mL of 80 mM 2-thiobarbituric acid was added. Samples were incubated in a water bath for 30 min and then in ice for 10 min. Absorbance was measured at 530 nm in a spectrophotometer (Perkin Elmer UV/VIS Spectrometer Lambda 2, Walluf, Germany). Thiobarbituric acid reactive substances (TBARS) values were reported in mg of malondialdehyde (MDA) per kg of meat, using a standard curve of MDA solution (Sigma Aldrich, Cat. No. 8057970050, MO).

### Quantitation of total volatile basic nitrogen (TVBN)

The quantification of TVBN was performed following the methodology reported by Chen^32^. Briefly, pork samples (25 g) were obtained and transferred to an Erlenmeyer flask with a ground glass stopper. 100 mL of water with some glass beads were placed and stirred for 30 min. Samples were filtered through filter paper Whatman type 4 and transferred into glass Petri dishes (edges were coated with petroleum jelly) and incubated at 40 °C for 3 h. A saturated solution of boric acid in glycerin (13 drops) and 2 mL of saturated sodium carbonate solution was placed on the internal face of the Petri dish lid. Both solutions were gently mixed. Samples were incubated at 40 °C for 3 h. Subsequently, drops that were formed under the glass cover were transferred to an Erlenmeyer flask with 60 mL of distilled water (pH 5.1), and 1 mL of methyl red, 0.5% (w/v) ethanol solution and 5 mL of bromocresol green 0.4% (w/v) alcoholic solution were added. Titration was performed with 0.01 N hydrochloric acid until a pink coloration was observed. TVBN values were calculated using Eq. (4) and expressed as mg of nitrogen per 100 g of pork meat:

4 $$\mathrm{TVBN}= \left(\frac{\mathrm{ V}*\mathrm{C}*14}{\mathrm{pork }(\mathrm{g})}\right)*100$$

where V is the added volume of HCl and C is the concentration of HCl.

### Statistical analyses

To determine differences among treatment and storage time means, Gaussian distribution was assessed and two-way analysis of variance (ANOVA) followed by Tukey’s HSD test were performed with a significance level of α = 0.05 (95% confidence). Correlation analysis for pH, content of lactic acid, TBARS and TVB-N was performed using the Pearson correlation coefficient. All analyses were performed using GraphPad Prism version 7.00 for Windows (GraphPad Software, La Jolla California USA, www.graphpad.com).

## Results and discussion

### Characteristics of used solutions

All the evaluated solutions were analyzed before use. The pH of NEW and NaClO were 6.92 ± 0.06 and 7.36 ± 0.28 respectively. SS showed a pH of 5.71 ± 0.3. NEW had the highest ORP value (820 ± 9.3 mV) followed by NaClO (790 ± 20.19 mV), and SS had the lowest value (371 ± 14.76 mV). NEW had a free chlorine value of 58 ± 2 ppm. The main component of NEW is hypochlorous acid; however, there are other components (~ 5%) like hypochlorite ions and trace amounts of chlorine with less presence^33^. NEW concentration was not adjusted because it has been reported that bactericidal activity is determined by chlorine concentration instead of the reaction time^5^. NaClO concentration was adjusted to 35 ppm with distillated water because this concentration is permitted by the USDA for use with other types of meats^34^ without affecting meat physicochemical properties. The concentration of free chlorine in SS was below our detection method. All results are summarized in Table 1.

**Table 1:** Physicochemical characteristics of solutions.

	pH	ORP (mV)	Free chlorine (ppm)
NEW	6.92 ± 0.06	820 ± 9.30	58.0 ± 2.0
NaClO	7.36 ± 0.28	790 ± 20.19	35.0 ± 0.59
SS	5.71 ± 0.30	371 ± 14.76	ND^a^

Values represent the mean ± SEM (n = 3).

^a^Not detectable.

### In vitro* bactericidal effect*

The bactericidal effect of NEW and NaClO were compared to the results obtained from SS. Both solutions caused a decrease in the *L. monocytogenes* numbers of > 4.9 log CFU/mL and *Salmonella* Typhi counts of > 6.3 log CFU/mL when in vitro values were compared with the SS treatment (wash control). NaClO and NEW treatments were not statistically different (P > 0.05). However, both groups were significantly different to the SS treatment (P < 0.0001) when NEW and NaClO were used with both bacteria. Nevertheless, NaClO and NEW bacterial bacteria values were lower than 3 log CFU/mL since we could not detect any growth beyond the methodology detection limit (Table 2). These results were consistent when 3-mL aliquots from each treatment were plated on three different TSA petri dishes (1 mL/plate).

**Table 2:** Surviving bacterial populations after treatment.

Type of evaluation	Units	SS	NEW^3^	NaClO^3^
*L. monocytogenes* (in vitro)	*Log CFU/mL*	7.93 ± 0.024 ^a^	< 3^b^	< 3^b^
In situ (pork + *L. monocytogenes*) (24 h after treatment)	*Log CFU/g*	5.52 ± 0.035^a^	4.883 ± 0.085^b^	4.899 ± 0.064^b^
In situ (pork + *L. monocytogenes*) (8 days after treatment)	*Log CFU/g*	7.945 ± 0.03^a^	6.885 ± 0.06^b^	6.777 ± 0.112^b^
*Salmonella* Typhi (in vitro)	*Log CFU/mL*	9.3 ± 0.03 a	< 3^b^	< 3^b^
In situ (pork + *Salmonella* Typhi) (24 h after treatment)	*Log CFU/g*	5.12 ± 0.21^a^	4.65 ± 0.27^a^	4.82 ± 0.27^a^
In situ (pork + *Salmonella* Typhi) (8 days after treatment)	*Log CFU/g*	5.29 ± 0.33^a^	5.51 ± 0.14^a^	5.09 ± 0.34^a^
Total aerobic viable counts on pork (24 h after treatment)	*Log CFU/g*	6.00 ± 0.11^a^	5.74 ± 0.12^a^	5.67 ± 0.08^a^
Total aerobic viable counts on pork (8 days after treatment)	*Log CFU/g*	7.26 ± 0.05^a^	7.19 ± 0.11^a^	7.32 ± 0.09^a^

Values represent the means ± SEM within a row without a common superscript are statistically significantly different (P < 0.0001).

### Bactericidal effect on contaminated pork chops

All treatments were applied by spraying because it uses less disinfectant solution than soaking the samples. NaClO and NEW treatments caused a reduction of *Listeria monocytogenes* counts by 0.621 and 0.637 log CFU/g on contaminated and treated pork chops. NaClO and NEW treatments were not statistically different (P < 0.05), but both were significantly different from the SS group (P < 0.0001). Contaminated pork with *Salmonella* Typhi showed reduction counts by 0.47 and 0.3 log CFU/g when NEW and NaClO treatments were used however, no significant difference was detected when results were compared with SS treatment. It has been reported that treatments combined with shaking methodologies improve the sanitization process^12,35–37^; this could remove surface bacteria and allow NEW to come into contact with deeper cells.

Treated pork chops were kept at 4 °C for 8 days. Bacterial counts for surviving bacteria are shown in Table 2. We detected that survival *L. monocytogenes* counts from treated pork were 1.168 and 1.06 log CFU/g lower for NaClO and NEW respectively compared to the SS treatment. NaClO and NEW were not statistically different but both were significantly different from the SS treatment (P < 0.0001). Bactericidal values were higher at day 8 than at day 1 (P < 0.0001). Similar results were reported when acid EW (pH 2.79) was used for 15 s in fresh pork contaminated with *L. monocytogenes* causing a decreasing titer of 1.39 or 0.56 log_10_ CFU/cm^2^ when they compared the effect with untreated or distilled water treatment respectively^38,39^. Similar bacterial reduction results were obtained in other studies^40,41^ where pork samples were immersed in EW and during the storage time, bacterial titers of pork dipped in EW were lower than control treatments. NEW and NaClO treated contaminated pork with *Salmonella* Typhi showed lower bacterial numbers by 0.47 or 0.3 respectively compared to the SS group after 60 s treatment nonetheless, there was no significant difference (Table 2) in bacterial reduction numbers between groups. Readouts at day 8 did not show significant reduction counts of *Salmonella* Typhi in three evaluated groups. This limited bactericidal efficacy is similar to a study where neutral EW was evaluated on pork and skin samples^42^. Other studies^5^ evaluated the effect of a similar neutral EW against *E. coli* O157:H7, *Salmonella* Enteritidis and *Yersinia enterocolitica* showing similar results to our in vitro experiment but with the use of flow cytometry technique. Nonetheless, in this study, reported *E. coli* reduction titers were 2.12 log CFU/cm^2^ and 2.22 log CFU/cm^2^ for *Salmonella* Enteritidis after two minutes of treatment. Additional studies reported the use of acidic EW (pH 2.6) for 40 s and the use of this solution caused the reduction of mesophilic bacteria on pork loins by 1.67 and 0.48 log CFU/g after 1 and 15 days of treatment (respectively)^20^. Our treatment was performed for 60 s and based on a previous report^43^, the longer the EW is in contact with pork the better the antibacterial effect expected. Nonetheless, the use of NEW or NaClO showed different bactericidal efficacies when solutions were evaluated against different bacteria; NEW demonstrated a better bactericidal effect against *L. monocytogenes* than against *Salmonella* Typhi; this result is similar to evaluations reported by Feliciano^44^ where slightly acidic EW was evaluated against *Listeria innocua*. NEW antibacterial activity could decrease the contamination of meat with other gram-positive pathogens like *Bacillus cereus*, *Lactobacillus* spp., *Lactococcus lactis*, or *Staphylococcus aureus*. In this study we used pork chops contaminated with *L. monocytogenes* reaching a titer of Log 7.9 CFU/g, this concentration is higher to those titers reported in contaminated pork products (10 CFU/g)^45^.

Total aerobic viable counts after 60 s treatment showed a bacterial decrease of 0.26 and 0.33 log CFU/g on non-contaminated pork, these values are not significant different with numbers from SS group; neither bacterial counts after 8 day storage showed difference with wash control group (Table 2). Our results suggest that the presence of organic matter on pork limits the efficacy of NEW against *Salmonella* Typhi and mesophilic bacteria. In our study, NEW was applied using spray bottles however, different studies^42,46^ showed better results when samples were dipped.

### Color measurements

The color of meat is the first visual parameter that consumers consider^47^; this is an important parameter in a disinfection process. Meat color is affected by the amount of myoglobin and its different forms like oxi-myoglobin and deoxymyoglobin, that are present^48^. Parameters *L*, *a,* and *b* from the CIE*Lab* spectra were measured (Table 3).

**Table 3:** Color score using the CIELAB space of pork chops.

Parameter	Day	Treated		
		NEW	NaClO	SS
*L*	1	58.20 ± 0.65^Ba^	58.84 ± 0.59^ABa^	62.28 ± 0.60^Aa^
	3	58.34 ± 1.68^Aa^	60.00 ± 1.72^Aa^	62.10 ± 1.58^Aa^
	5	57.42 ± 2.96^Aa^	60.56 ± 1.12^Aa^	59.70 ± 1.18^Aa^
	12	55.66 ± 1.04^Baa^	60.72 ± 1.52^Aa^	58.48 ± 1.84^ABa^
	19	55.91 ± 1.19^Aaa^	58.36 ± 0.52^Aa^	59.42 ± 0.70^Aa^
*a*	1	6.29 ± 0.26^Aab^	1.91 ± 0.14^Bb^	1.77 ± 0.17^Bb^
	3	5.62 ± 0.41^Ab^	2.64 ± 0.29^Bbc^	2.20 ± 0.45^Bb^
	5	6.42 ± 0.75^Aab^	3.40 ± 0.22^Bacd^	4.16 ± 0.54^Ba^
	12	6.6 ± 0.20^Aab^	3.21 ± 0.35^Bbde^	5.34 ± 0.51^Aa^
	19	7.23 ± 0.38^Aa^	4.44 ± 0.44^Bae^	5.42 ± 0.19^Ba^
*b*	1	12.38 ± 0.27^Ab^	11.71 ± 0.37^Ab^	12.17 ± 0.20^Ab^
	3	13.01 ± 0.47^Aab^	12.34 ± 0.45^Aab^	12.92 ± 0.50^Aab^
	5	13.54 ± 0.76^Aab^	12.56 ± 0.26^Aab^	13.33 ± 0.45^Aab^
	12	13.78 ± 0.51^Aab^	13.28 ± 0.35^Aa^	13.43 ± 0.59^Aab^
	19	14.12 ± 0.34^Aa^	13.67 ± 0.27^Aa^	14.15 ± 0.31^Aa^

Values represent the means ± SEM.

^a^^—^^c^Significant difference within each column (P < 0.05).

^A^^—^^B^Significant difference within each row (P < 0.05).

*L* values from treated pork were different between NEW and SS groups at day 1. At days 3, 5, and 19, we did not detect significant difference between groups, but at day 12 NaClO and NEW were statistically different (P < 0.05). *L* values from each treatment did not change by the time (P > 0.05). The formation of the dark color in meat has been related to the accumulation of metmyoglobin. This is formed by the oxidation of iron in the protein from the ferrous to ferric state^49^.

Another analyzed parameter was the *a* value (Table 3). When NEW was used, *a* values increased from day 5 to 19. However, there were not significant differences between days (P > 0.05). At the same time, NEW generated the highest values between treatments. NaClO and SS groups showed an increase in *a* values by the time increases (P < 0.0001).

The last evaluated color parameter was the *b* value. Conversely, all treatments were similar, and the only difference was detected at day 19 (P < 0.05).

**Figure 1:**
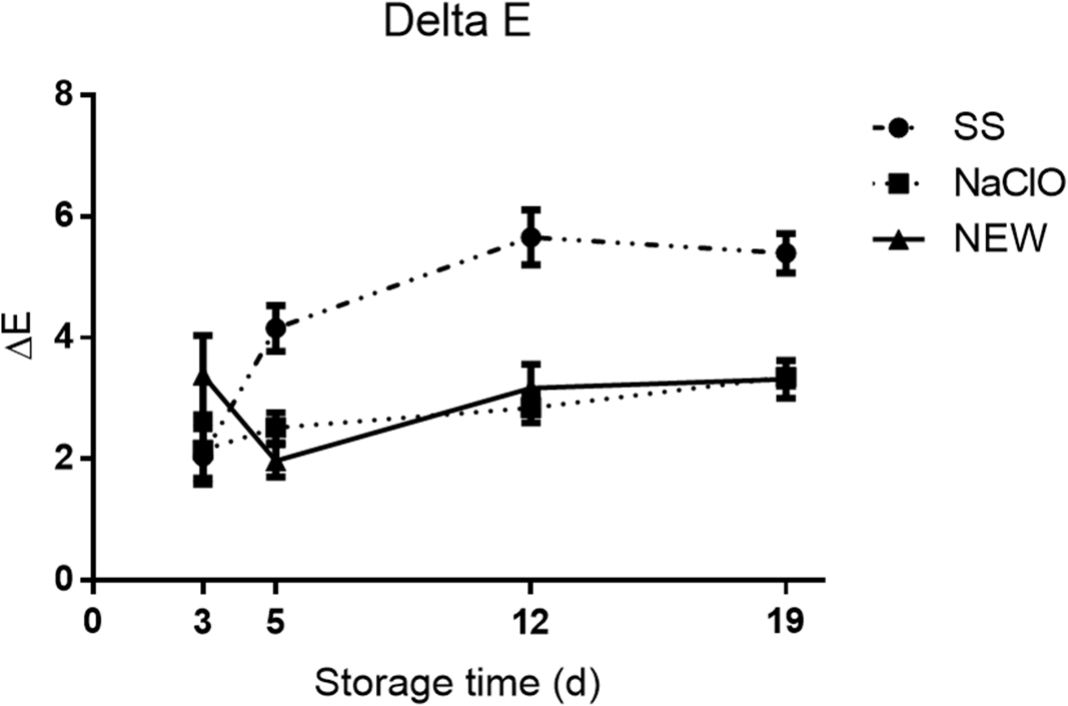
Delta E values. Effect on the global color change using disinfectant solutions on pork chops. Measurements were performed on indicated days using 5 random areas/sample. Values represent the mean ± standard error (SEM), (P < 0.005).

Delta E was calculated to detect which treatment caused a higher change in pork chop color. Pork treated with SS had the most color changes while NEW and NaClO groups had less (Fig. 1). NEW and NaClO showed similar values. Delta E values from SS and NaClO groups at day 3 were similar (P < 0.05). Nonetheless, from day 5 to 19, NEW and NaClO groups were significantly lower than the SS group. Both treatments (NEW and NaClO) helped to avoid color changes when pork was kept in refrigeration. Phosphate-enhanced (alkaline electrolyzed water) has been used on steaks^50^ and meat kept a moderately red color after 14 days of storage at 4 °C. There are not many studies about the color change and the use of EW. This quantitative parameter can provide an additional characteristic to additives or ingredients that are used in the meat process. Color is a parameter that all customers can detect easily and could drives the customer decision to buy the final product and is related to shelf live.

### pH and lactic acid

Pork was treated with NEW and NaClO solutions that had a neutral pH while saline solution had a pH of 5.71 (Table 1). The inner characteristics of each solution could affect pork characteristics like water retention. The pH of treated pork was analyzed, and we did not detect any differences from day 1 to 19 (Table 4). The pH of meat treated with SS and NEW did not change with time. However, there is a positive correlation between pH and *a* (R^2^ = 0.78), and pH and *b* (R^2^ = 0.905) in chops treated with SS; we interpreted this information as with time, the non-disinfected pork lowers its pH and increases it red and yellow characteristics. These phenomena did not exist when pork was treated with NaClO. In the pork treated with NEW, pH values were positively correlated with *L* (R^2^ = 0.85) and *a* values (R^2^ = 0.864); this data suggests that the use of NEW generates the appearance of a darker, redder color while at the same time the pH is lowering. Nonetheless, NaClO treatment caused a decrease at days 19 (P < 0.01) but no statistical difference was detected between treatments (P > 0.05). These results suggest that NEW reacted on bacterial cells without affecting meat’s pH. Similar results were reported previously when slightly acidic EW was used alone or in combination with basic EW^20^.

**Table 4:** pH of pork chops.

Day	Treatment		
	NEW	NaClO	SS
1	5.7 ± 0.026^Aab^	5.71 ± 0.012^Aa^	5.69 ± 0.022^Aa^
3	5.79 ± 0.028^Ab^	5.69 ± 0.004^ABa^	5.672 ± 0.026^Bab^
5	5.75 ± 0.072^ABab^	5.78 ± 0.004^Aa^	5.682 ± 0.023^Bab^
12	5.67 ± 0.014^Aa^	5.65 ± 0.013^Aac^	5.61 ± 0.023^Aab^
19	5.63 ± 0.036^Aa^	5.54 ± 0.044^Abc^	5.57 ± 0.009^Ab^

Values represent the means ± SEM.

^A^^—^^B^Significant difference within each row (P < 0.05).

^a^^—^^c^Significant difference within each column (P < 0.05).

**Figure 2:**
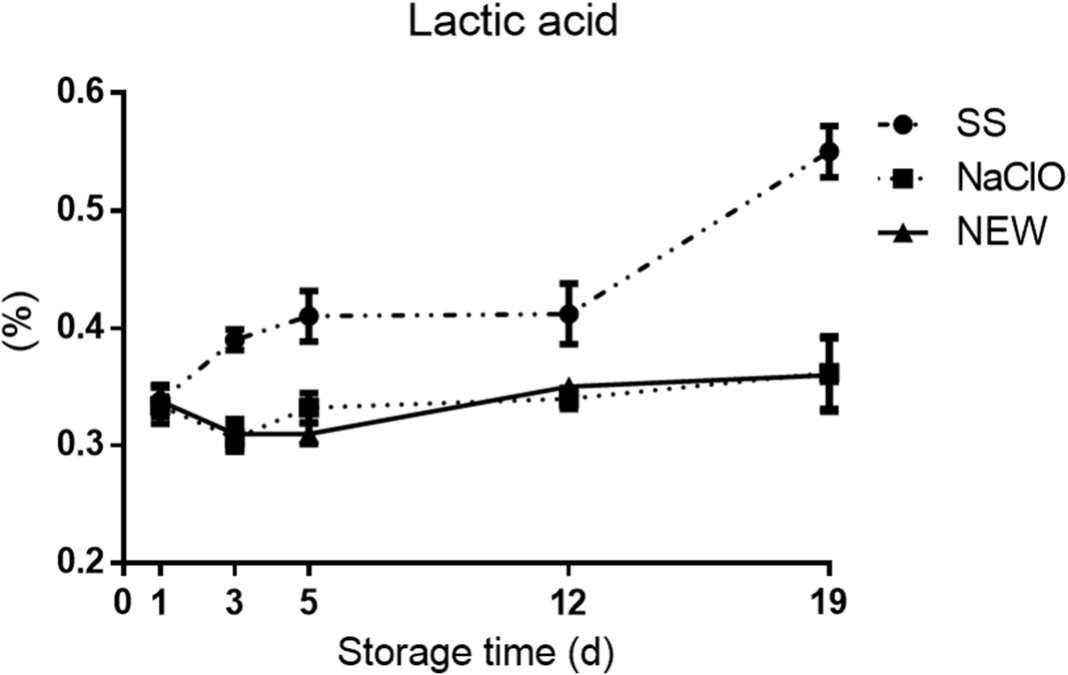
Lactic acid presence (as percentage) in treated samples. Pork chops were treated with evaluated solutions and kept at refrigeration temperature. Values represent the mean ± SEM, (P < 0.005).

During meat processing, oxygen concentration decreases. This change causes lactic acid to accumulate and pH decreases from neutral to a range of 5.7–5.3^51^. For all treatments, we detected that pH declined with the time and the concentration of lactic acid increases at the same time (Table 4 and Fig. 2). The concentration of lactic acid at day 1 was similar in all groups, however from day 5 to 19, SS treatment showed a significant increase (P < 0.05) (Fig. 2). We detected two important days where the increase of lactic acid occurs; the first is between day 3 and 5 and the second important increase is at day 12. Nonetheless, NaClO and NEW treatments showed a similar lactic acid amount from days 3 to 19 and were statistically different from the SS group (P < 0.01). The increase of lactic acid in the SS group could have happened because of the presence of fast-growing bacteria that generate lactic acid during the pork acquisition. That would explain the lower levels in the NaClO and NEW treatments.

#### TBARS

The thiobarbituric acid reactive substances (TBARS) assay was performed to quantify the amount of secondary lipid oxidation products in pork chops after treatment^52^. TBARS values (Table 5) were increasing for all treatments as time elapsed up to day five. NEW and NaClO treatments showed higher values than the SS treatment; this effect could be due to its higher ORP values (Table 1). The ORP of NEW and NaClO solutions could cause an oxidation of fatty acids which could continue during storage. For all the groups, the generation of TBARS stabilized at day 5. Connel established a threshold of 2 mg MDA/kg for human consumption^53^, nevertheless, 0.5 mg MDA/kg has been the threshold for the detection of off-flavors^54–56^ and all treatments were below this level. This could be as a result of the combination of NEW or NaClO with the low content of polyunsaturated fatty acids in pork meat and the storage at 4 °C which produce less MDA concentration^57^. A similar study was performed earlier^20^ where pork was treated with slightly acidic EW however, results stablished that the use of EW did not accelerate the lipid oxidation in pork loins.

**Table 5:** Lipid oxidation (expressed in TBARS) in treated pork chops.

Day	Treated		
	NEW	NaClO	SS
1	0.230 ± 0.006^Ab^	0.164 ± 0.028^Bb^	0.142 ± 0.009^Bb^
3	0.287 ± 0.018^Abc^	0.198 ± 0.022^Bb^	0.190 ± 0.006^Bb^
5	0.391 ± 0.003^Aa^	0.338 ± 0.006^Aa^	0.379 ± 0.015^Ac^
12	0.354 ± 0.006^Aa^	0.329 ± 0.038^ABa^	0.279 ± 0.007^Ba^
19	0.350 ± 0.013^Aac^	0.327 ± 0.009^ABa^	0.267 ± 0.019^Ba^

Values represent the means ± SEM.

^a^^—^^e^Significant difference within each column (P < 0.05).

^A^^—^^B^Significant difference within each row (P < 0.05).

### Total volatile basic nitrogen

**Figure 3:**
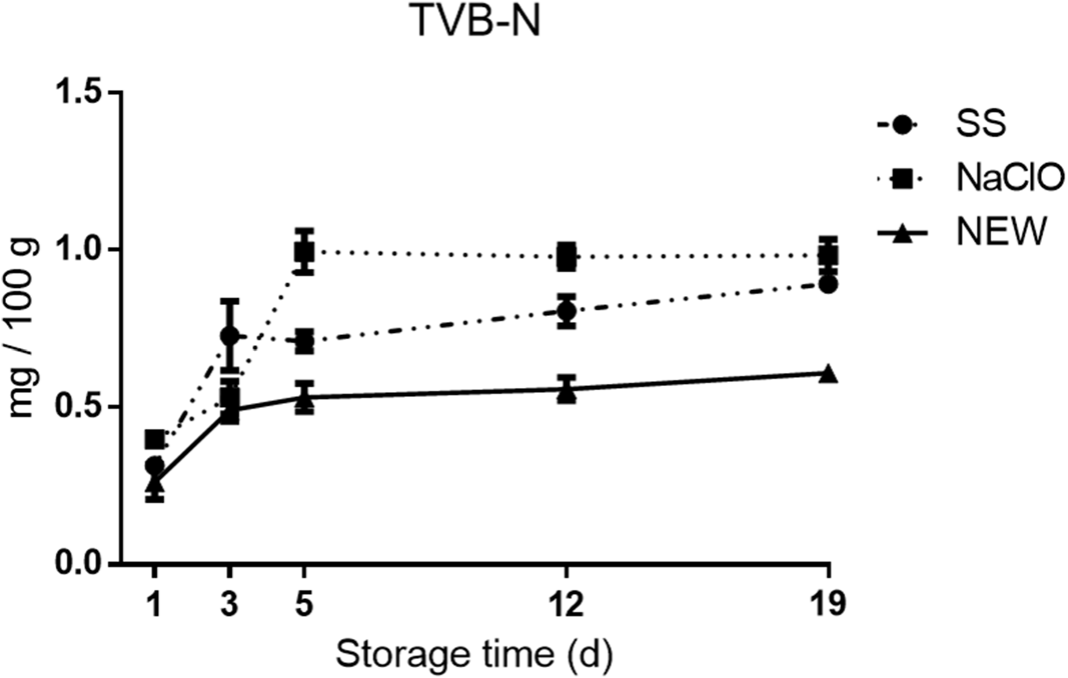
TVB-N present on pork chops after treated with SS, NaClO or NEW. Values represent the means ± SEM. (P < 0.001).

The calculation of Total Volatile Basic Nitrogen (TVB-N) is a common procedure^58^ that is related with meat freshness^59^. Intensification of dimethylamine (generated by lytic enzymes during storage) and ammoniac (generated by deamination of amino acids and nucleotide catabolism) is an indicator of bacterial contamination^58,60^. For all treatments, the TVB-N values increased with respect to the storage time (Fig. 3); reaching the higher concentration values at day 19. When we compare the generation of TVB-N in every collection day, we detected that at day 1 all treatments were similar (P > 0.05) however, at day 3, SS treatment was statistically different (P < 0.05) from NaClO and NEW treatments and, at days 5 and 12 all treatments were significantly different (P < 0.05) from each other. Finally, at day 19, NEW treatment was different from the rest of treatments (P < 0.005) nevertheless, NEW generate a low constant concentration of TVB-N in all the time points. These findings suggest that NEW significantly slows the decomposition of pork chops generating important reagents that affect the quality of meat. Cadaverine and putrescine are the main biogenic amines that are produced when pork is stored at 4 °C^59^. When we relate TVB-N with pH, we detected that pH values are positively associated with TVB-N when the chops are treated with SS (no disinfection) (R^2^ = 0.8479), and that this association is blocked when pork is treated with NEW or NaClO. This phenomena is caused because bacteria break down meat proteins, generating basic compounds^61,62^.

## Conclusion

Pork is one of the most consumed agricultural products in the world. Some of the foodborne outbreaks have been related to the consumption of contaminated pork with *L. monocytogenes*^63,64^. NEW showed a high antibacterial activity against *L. monocytogenes *in vitro (as a pure culture) and in highly contaminated pork chops. The use of NEW in meat processing could help to eliminate bacterial contamination. Its effects also help during storage at 4 °C. Its antibacterial property is comparable to sodium hypochlorite. NEW does not affect the content of TVB-N, pH, meat color and lactic acid production was low. Neutral Electrolyzed Water increased lipid oxidation without reaching the limit for human consumption. The presence of organic could compromise NEW bactericidal activity nevertheless, NEW could be an effective alternative to decontaminate pig meat, carcasses and to disinfect meat-processing plants however, further studies are needed on different types of application (spray vs dip) and time.

## References

[1] Food and Agriculture Organization (FAO). FAOSTAT. (2018). https://www.fao.org/faostat/en/#data/QL/visualize. (Accessed 20 November 2018).

[2] Sohaib M, Anjum FM, Arshad MS, Rahman UU (2016). Postharvest intervention technologies for safety enhancement of meat and meat based products; a critical review. J. Food Sci. Technol..

[3] Fallik E, Florkowski WJ, Shewfelt RL, Brueckner B, Prussia SE (2014). Microbial quality and safety of fresh produce. Postharvest Handling. A system Approach.

[4] Bilek SE, Turantaş F (2013). Decontamination efficiency of high power ultrasound in the fruit and vegetable industry, a review. Int. J. Food Microbiol..

[5] Han D, Hung Y, Wang L (2018). Evaluation of the antimicrobial ef fi cacy of neutral electrolyzed water on pork products and the formation of viable but nonculturable (VBNC) pathogens. Food Microbiol..

[6] Jayathilakan K, Sharma GK, Radhakrishna K, Bawa AS (2007). Antioxidant potential of synthetic and natural antioxidants and its effect on warmed-over-flavour in different species of meat. Food Chem..

[7] Satyanarayan VT, Honikel KO (1992). Effect of different cooking methods on warmed-over flavour development in pork. Z. Lebensm. Unters. Forsch..

[8] Meireles A (2016). An Outbreak of human listeriosis in England between 2010 and 2012 associated with the consumption of pork pies. Int. J. Food Microbiol..

[9] Centers for Disease Control and Prevention. CDC food safety alert: Outbreak of listeria infections linked to pork products produced by long phung food products. *CDC Newsroom releases* (2018). Available at: https://www.cdc.gov/media/releases/2018/s1121-Listeria-outbreak-linked-to-pork.html.

[10] Awofisayo-Okuyelu A (2016). An outbreak of human listeriosis in England between 2010 and 2012 associated with the consumption of pork pies. J. Food Prot..

[11] Afari GK, Hung YC, King CH (2015). Efficacy of neutral pH electrolyzed water in reducing *Escherichia coli* O157:H7 and Salmonella Typhimurium DT 104 on fresh produce items using an automated washer at simulated food service conditions. J. Food Sci..

[12] Guentzel JL, Lam KL, Callan MA, Emmons SA, Dunham VL (2008). Reduction of bacteria on spinach, lettuce, and surfaces in food service areas using neutral electrolyzed oxidizing water. Food Microbiol..

[13] Hung YC, Bailly D, Kim C, Zhao Y, Wang X (2010). Effect of electrolyzed oxidizing water and chlorinated water treatments on strawberry and broccoli quality. J. Food Qual..

[14] Morita C, Nishida T, Ito K (2011). Biological toxicity of acid electrolyzed functional water: Effect of oral administration on mouse digestive tract and changes in body weight. Arch. Oral Biol..

[15] Ayebah B, Yen-Con H (2005). Electrolyzed water and its corrosiveness on variuos surface materials commonly found in food processing facilities. J. Food Process Eng..

[16] Rivera-Garcia A (2019). The effect of neutral electrolyzed water as a disinfectant of eggshells artificially contaminated with *Listeria monocytogenes*. Food Sci. Nutr..

[17] Huang YR, Hung YC, Hsu SY, Huang YW, Hwang DF (2008). Application of electrolyzed water in the food industry. Food Control.

[18] Al-Haq MI, Sugiyama J, Isobe S (2005). Applications of electrolyzed water in agriculture and food industries. Food Sci. Technol. Res..

[19] Rahman SME, Park J, Song KB, Al-Harbi NA, Oh DH (2012). Effects of slightly acidic low concentration electrolyzed water on microbiological, physicochemical, and sensory quality of fresh chicken breast meat. J. Food Sci..

[20] Rodrigues-Athayde D (2017). Application of electrolyzed water for improving pork meat quality. Food Res. Int..

[21] Jardon-Xicotencatl S, Díaz-Torres R, Marroquín-Cardona A, Villarreal-Barajas T, Méndez-Albores A (2015). Detoxification of aflatoxin-contaminated maize by neutral electrolyzed oxidizing water. Toxins (Basel)..

[22] Rico D (2008). Use of neutral electrolysed water (EW) for quality maintenance and shelf-life extension of minimally processed lettuce. Innov. Food Sci. Emerg. Technol..

[23] Deza MA, Araujo M, Garrido MJ (2003). Inactivation of *Escherichia coli* O157:H7, *Salmonella enteritidis* and *Listeria monocytogenes* on the surface of tomatoes by neutral electrolyzed water. Lett. Appl. Microbiol..

[24] Rahman SME, Jin YG, Oh DH (2010). Combined effects of alkaline electrolyzed water and citric acid with mild heat to control microorganisms on cabbage. J. Food Sci..

[25] Comisión Federal para la Protección contra Riesgos Sanitarios. NOM-210-SSA1-2014, Productos y servicios. Métodos de prueba microbiológicos. Determinación de microorganismos indicadores. Determinación de microorganismos patógenos.* Diario Oficial de la Federación* (2014).

[26] Secretaría de Salud. NOM-092-SSA1-1994. Método para la cuenta de bacterias aerobias en placa. *Diario Oficial de la Federación* (1994).

[27] Food Safety and Inspection Service. *Table of Safe and Suitable Ingredients: Antimicrobial Update 5/25/2017* (2017).

[28] Greenberg AE, APHA/AWWA/WEF (2012). Iodometric method I. Standard Methods for the Examination of Water and Wastewater.

[29] SE, S. de C. y N./S. de E. NMX-F-317-NORMEX-2013 Alimentos-Determinación de pH en alimentos y bebidas no alcohólicas-Método Potenciométrico-Método de Prueba. *Diario Oficial de la Federación* (2013).

[30] SE, S. de C. y N / S de E. NMX-F-102-NORMEX-2010, Alimentos-Determinación de acidez titulable en alimentos-Método de ensayo.* Diario Oficial de la Federación* (2010).

[31] Hernández-Hernández E (2019). Microbiological and physicochemical properties of meat coated with microencapsulated Mexican Oregano (*Lippia graveolens* Kunth) and Basil. Coatings.

[32] Chen J, Xu B, Deng S, Huang Y (2016). Effect of combined pretreatment with slightly acidic electrolyzed water and botanic biopreservative on quality and shelf life of Bombay Duck (*Harpadon nehereus*). J. Food Qual..

[33] Liao LB, Chen WM, Xiao XM (2007). The generation and inactivation mechanism of oxidation-reduction potential of electrolyzed oxidizing water. J. Food Eng..

[34] United States Department of Agriculture. Related Documents for FSIS Directive 7120.1—Safe and Suitable Ingredients used in the Production of Meat, Poultry, and Egg Products. (2018). https://www.fsis.usda.gov/wps/portal/fsis/topics/regulations/directives/7000-series/safe-suitable-ingredients-related-document. (Accessed 25 November 2018)

[35] Park H, Hung Y-C, Brackett RE (2002). Antimicrobial effect of electrolyzed water for inactivating *Campylobacter jejuni* during poultry washing. Int. J. Food Microbiol..

[36] Vandekinderen I (2009). Effect of decontamination on the microbial load, the sensory quality and the nutrient retention of ready-to-eat white cabbage. Eur. Food Res. Technol..

[37] Flores DRM (2017). The use of ultrasound and slightly acidic electrolyzed water as alternative technologies in the meat industry. Food Res..

[38] Fabrizio KA, Cutter CN (2004). Comparison of electrolyzed oxidizing water with other antimicrobial interventions to reduce pathogens on fresh pork. Meat Sci..

[39] Brychcy E, Malik M, Drozdzewski P, Ulbin-Figlewicz N, Jarmoluk A (2015). Low-concentrated acidic electrolysed water treatment of pork: Inactivation of surface microbiota and changes in product quality. Int. J. Food Sci. Technol..

[40] Rahman SME, Wang J, Oh DH (2013). Synergistic effect of low concentration electrolyzed water and calcium lactate to ensure microbial safety, shelf life and sensory quality of fresh pork. Food Control.

[41] Mansur AR, Oh DH (2015). Combined effects of thermosonication and slightly acidic electrolyzed water on the microbial quality and shelf life extension of fresh-cut kale during refrigeration storage. Food Microbiol..

[42] Han D, Hung YC, Wang L (2018). Evaluation of the antimicrobial efficacy of neutral electrolyzed water on pork products and the formation of viable but nonculturable (VBNC) pathogens. Food Microbiol..

[43] Zhao Y (2014). Free chlorine loss during spraying of membraneless acidic electrolyzed water and its antimicrobial effect on airborne bacteria from poultry house. Ann. Agric. Environ. Med..

[44] Feliciano L, Lee J, Pascall MA (2012). Transmission electron microscopic analysis showing structural changes to bacterial cells treated with electrolyzed water and an acidic sanitizer. J. Food Sci..

[45] Thévenot D, Dernburg A, Vernozy-Rozand C (2006). An updated review of *Listeria monocytogenes* in the pork meat industry and its products. J. Appl. Microbiol..

[46] Khazandi M (2017). Efficacy evaluation of a new water sanitizer for increasing the shelf life of Southern Australian King George Whiting and Tasmanian Atlantic Salmon fi llets. Food Microbiol..

[47] Khliji S, van de Ven R, Lamb TA, Lanza M, Hopkins DL (2010). Relationship between consumer ranking of lamb colour and objective measures of colour. Meat Sci..

[48] Giddings GG, Solberg M (1977). The basis of color in muscle foods. Crit. Rev. Food Sci. Nutr..

[49] Mikkelsen A, Juncher D, Skibsted LH (1999). Metmyoglobin reductase activity in porcine *M. longissimus* dorsi muscle. Meat Sci..

[50] Stetzer AJ, Tucker E, McKeith FK, Brewer MS (2007). Quality changes in beef gluteus medius, infraspinatus, psoas major, rectus femoris, and teres major enhanced prior to aging. J. Food Sci..

[51] Ponce-Alquicira, E., Braña-Valera, D., López-Hernández, L. H. & Delgado-Suárez, E. Escalas descriptivas para la evaluación del color de la carne de cerdo, res y pollo. In *Evaluación de la frescura de la carne* (ed. Braña-Varela, D.) 80–81 (Instituto Nacional de Investigaciones Forestales, Agrícolas y Pecuarias, 2013).

[52] Hu J, Wang X, Xiao Z, Bi W (2015). Effect of chitosan nanoparticles loaded with cinnamon essential oil on the quality of chilled pork. LWT Food Sci. Technol..

[53] Connell, J. J. Methods of assessing and selecting for quality. In *Control of Fish Quality* (ed. Connel, J. J.) 256 (Wiley, 1990).

[54] Cerruto-Noya CA, Vanoverbeke DL, Dewitt CAM (2009). Evaluation of 0.1% ammonium hydroxide to replace sodium tripolyphosphate in fresh meat injection brines. J. Food Sci..

[55] Lanari MC, Schaefer DM, Scheller KK (1995). Dietary vitamin E supplementation and discoloration of pork bone and muscle following modified atmosphere packaging. Meat Sci..

[56] Lauzurica S (2005). Effect of dietary supplementation of vitamin e on characteristics of lamb meat packed under modified atmosphere. Meat Sci..

[57] Wenjiao F, Yongkui Z, Yunchuan C, Junxiu S, Yuwen Y (2014). TBARS predictive models of pork sausages stored at different temperatures. Meat Sci..

[58] Vinci G, Antonelli ML (2002). Biogenic amines: Quality index of freshness in red and white meat. Food Control.

[59] Min JS (2007). Relationship between the concentration of biogenic amines and volatile basic nitrogen in fresh beef, pork, and chicken meat. Asian-Austr. J. Anim. Sci..

[60] Min JS, Lee SO, Jang A, Lee M, Kim Y (2004). Quantitative analysis of biogenic amines in raw and processed foods of animal origin on Korean domestic market. Asian-Austr. J. Anim. Sci..

[61] Mansour AFA, Zayed AF, Basha OLAAA (2015). Contamination of the shell and internal content of table eggs with some pathogens during different storage periods. Assiut Vet. Med. J..

[62] Tan W, Shelef LA (2002). Effects of sodium chloride and lactates on chemical and microbiological changes in refrigerated and frozen fresh ground pork. Meat Sci..

[63] Anonymous. Foodborne illness outbreak database. https://outbreakdatabase.com/site/search/?outbreak=&vehicle=&organism=Listeria+monocytogenes&month=&year=&state=0&country=US&x=22&y=22. (Accessed 6 June 2019)

[64] Centers for Disease Control and Prevention. National Outbreak Reporting System. https://wwwn.cdc.gov/norsdashboard/. (Accessed 6 April 2019).

